# Peroxidase Mimetic Nanozymes in Cancer Phototherapy: Progress and Perspectives

**DOI:** 10.3390/biom11071015

**Published:** 2021-07-11

**Authors:** Suresh Thangudu, Chia-Hao Su

**Affiliations:** 1 Institute for Translational Research in Biomedicine, Kaohsiung Chang Gung Memorial Hospital, Kaohsiung 833, Taiwan; suresh120689@gmail.com; 2 Department of Biomedical Imaging and Radiological Sciences, National Yang Ming Chiao Tung University, Taipei 112, Taiwan

**Keywords:** nanomaterials, enzyme mimetic, nanozymes, peroxidase mimetic, phototherapy, cancer therapy, theranostics, dual enzyme, single atom

## Abstract

Nanomaterial-mediated cancer therapeutics is a fast developing field and has been utilized in potential clinical applications. However, most effective therapies, such as photodynamic therapy (PDT) and radio therapy (RT), are strongly oxygen-dependent, which hinders their practical applications. Later on, several strategies were developed to overcome tumor hypoxia, such as oxygen carrier nanomaterials and oxygen generated nanomaterials. Among these, oxygen species generation on nanozymes, especially catalase (CAT) mimetic nanozymes, convert endogenous hydrogen peroxide (H_2_O_2_) to oxygen (O_2_) and peroxidase (POD) mimetic nanozymes converts endogenous H_2_O_2_ to water (H_2_O) and reactive oxygen species (ROS) in a hypoxic tumor microenvironment is a fascinating approach. The present review provides a detailed examination of past, present and future perspectives of POD mimetic nanozymes for effective oxygen-dependent cancer phototherapeutics.

## 1. Introduction

Cancer is one of the leading causes of human mortality [1]. Major problems associated with cancer treatment include the reoccurrence of tumors, tumor metastasis and resistance to chemo drugs [2,3]. Chemotherapy, radiotherapy (RT), and surgery are currently the most efficient treatment modalities, but have significant drawbacks including damage to normal cells, tumor reoccurrence, poor visualization and tumor hypoxia [4,5,6]. Therapeutic and diagnostic strategies for treating the cancer efficiently are needed. Recently, significant attention has been focused on nanomaterial-mediated phototherapies such as photothermal therapy (NmPTT) and photodynamic therapy (NmPDT) for the treatment of many diseases including cancers, bacterial infections, etc. [7,8,9,10,11,12]. NmPTT relies on photothermal heat and NmPDT mainly relies on reactive oxygen species (ROS). Compared to traditional therapeutic modalities, NmPDT is highly selectivity, can be remote controlled, has low systemic toxicity and is noninvasive [13,14]. In terms of its mechanism, PDT is strongly dependent on oxygen, and insufficient oxygen in the tumor microenvironment (tumor hypoxia) makes PDT less effective in in vivo systems [6]. Several factors contribute to tumor hypoxia, such as excess extracellular matrix, low pH values, and immunosuppressive factors resulting from altered metabolic pathways and abnormal tumor vasculature [15]. Several studies have found that tumor hypoxia promotes tumor growth [16,17]. Promising results have been achieved in addressing tumor hypoxia by supplying tumors with oxygen using oxygen carriers such as Hb oxygen carriers, non- Hb oxygen carriers and hybrid proteins, etc. [18,19]. However, limited loading efficiency and release of O_2_ on oxygen nanocarriers is still a limiting factor [20]. To this end, increased attention has been focused on generating O_2_ on nanomaterials, specifically on enzyme mimetic nanomaterials, known as “nanozymes”, to overcome tumor hypoxia and mediate cancer therapeutics [21]. Two different therapeutic approaches, direct killing (increasing ROS) and indirect killing (depletion of ROS) have been used to investigate cancer therapeutics using various nanozymes, mainly peroxidase (POD), oxidase (OXD), superoxide dismutase (SOD) and catalase (CAT) mimetic nanomaterials [22]. The approach of increasing ROS promotes therapeutic efficiency particularly with the use of POD mimetics by overcoming tumor hypoxia in oxygen-dependent PDT. POD mimetic nanozymes catalyze the endogenous H_2_O_2_ and produce H_2_O and ROS in the tumor microenvironment. However, a detailed review of POD mimetic nanozymes reports for cancer therapeutics is still lacking.

**Scheme 1:**
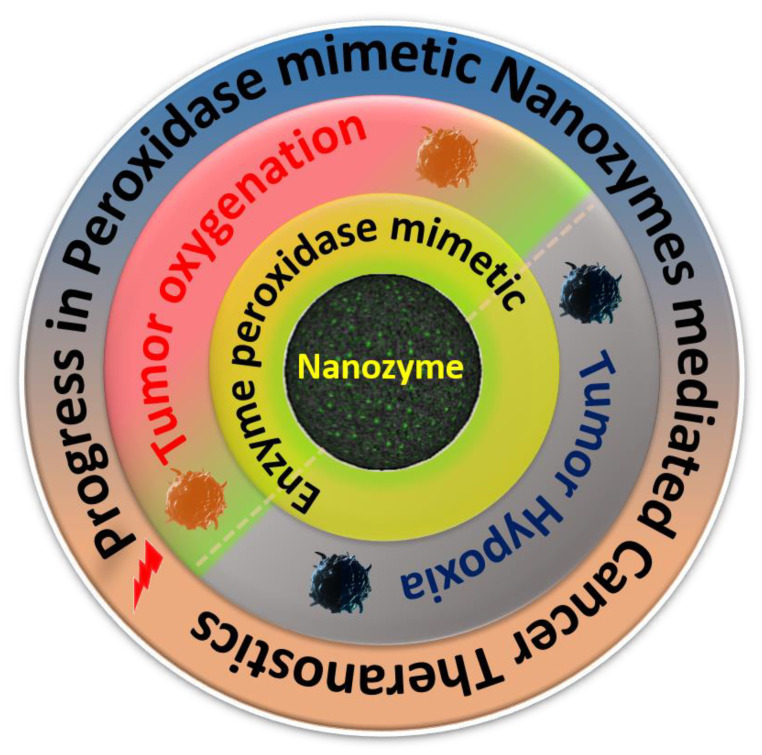
Graphical representation of the present review.

This review presents current advancements and future perspectives of the use of POD mimetic nanozymes in oxygen-dependent cancer PDT. A graphical summary of the present review is shown in Scheme 1.

## 2. Nanozymes

**Figure 1:**
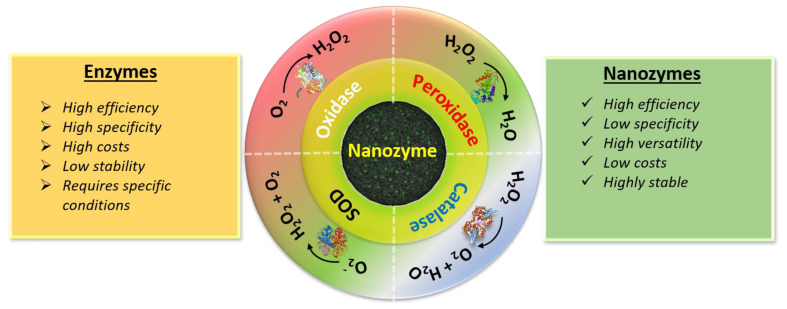
The properties of various nanozymes and their advantages over enzymes.

Enzymes can serve as biological catalysts (e.g., POD, OXD SOD and CAT, etc.) for various in vivo biological reactions [23]. Like all catalysts, enzymes have two fundamental properties: firstly, they increase the catalytic reaction without being consumed themselves, and secondly, they increase the reaction rates without altering the chemical equilibrium. In general, natural enzymes consist mainly of two parts: a protein and a metallic cofactor. The protein part contains various functional groups and facilitates absorption of the substrate and provides an active site for substrate binding, whereas the metallic part (generally metal ion or metallic complex) facilitates electron transmission. The simultaneous action of the two parts enhances the enzyme’s catalytic activity [24]. Due to superior catalytic activity and excellent substrate specificity, several enzymes have been used in various applications such as agrochemical production, pharmaceutical processes, food industry applications and biomedical applications [25,26,27]. However, the practical applications of enzymes are restricted due to some serious limitations. As shown in Figure 1, enzymes have disadvantages such as low operational stability, low sensitivity and high cost, etc. Thus, an alternative strategy to mimic natural enzymes and enhance catalytic reactions is urgently required.

Thus, considerable effort has been devoted to developing nanozymes which are similar to natural enzymes and can effectively catalyze the conversion of enzyme substrates under mild conditions, and exhibit similar catalytic efficiency and enzymatic reaction kinetics [28]. As shown in Figure 1, nanozymes exhibit several advantages over natural enzymes, including multifunctionality, tunability of catalytic activities, low cost, production scalability, recyclability and high stability. Moreover, these nanozymes can function in ambient conditions. Nanozyme activity can be tuned by simply varying their shape, structure, and composition, and considerable effort has focused on investigating the theoretical mechanisms and kinetics of nanozymes [29].

The advantages of nanozymes have led to their use in energy, environmental and biomedical applications. For instance, nanozymes can be used to qualitatively and quantitatively detect environmental toxins such as ions, molecules and organic compounds [30]. Nanozymes have also been used to treat bacterial infections, offering exciting broad-spectrum antimicrobial properties with negligible cytotoxicities [31]. They have also been used in biosensors for the rapid, reliable, and highly sensitive detection of various diseases [32,33]. Recently, nanomaterial-based nanozymes have been extensively investigated for use in the treatment of cancer through overcoming tumor hypoxia, since oxygen plays a pivotal role in cancer development and treatment [34]. Various enzyme mimetic nanomaterials, such POD OXD, SOD and CAT mimetic nanozymes, have been extensively studied for use in cancer therapeutics [34]. The present review mainly focuses on POD mimetic nanomaterials in cancer theranostic applications, and offers a detailed discussion of current advances and future perspectives.

### 2.1. Peroxidase Mimetic Nanozymes: Mechanisms and Role

Peroxidase is a natural enzyme found in wide variety of organisms, from plants to humans to bacteria [35]. The main function of the peroxidase enzyme is the decomposition of H_2_O_2_ to nontoxic components. H_2_O_2_ is a toxic byproduct formed by respiration of O_2_ [36]. Peroxide enzymes act as detoxifying agents for free radicals (e.g., glutathione peroxidase) and also aid defense against invading pathogens (e.g., myeloperoxidase). Peroxidases are also widely used in bioanalytical and clinical chemistry applications for the detection of analytes via colorimetric assays. As mentioned earlier, to overcome the drawbacks of natural enzymes, significant effort has gone into developing effective alternative POD mimetic enzymatic strategies.

**Figure 2:**
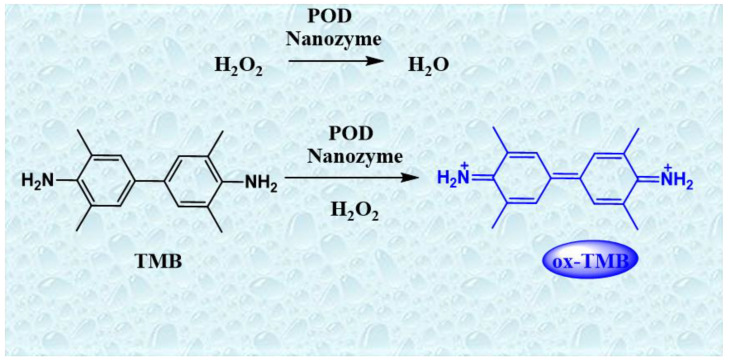
Schematic representation of POD mimetic assay.

**Figure 3:**
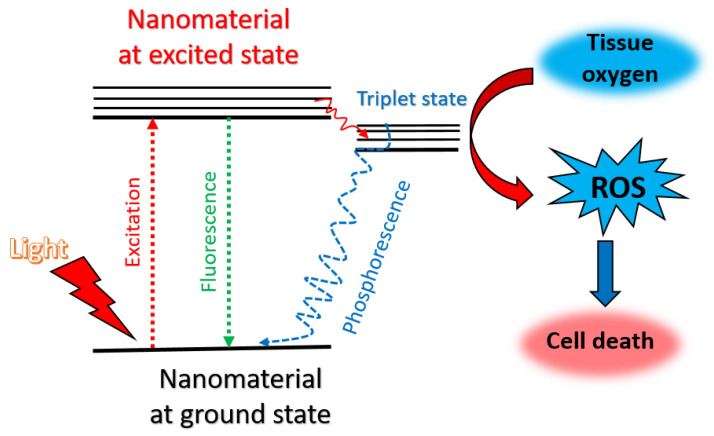
Mechanism of nanomaterials mediated photodynamic therapy.

The first evidence of Fe_3_O_4_ nanoparticles as peroxidase mimetic was reported in 2007 [37]. Several Fe-based nanocomposites have been found to exhibit activity very similar to that of natural horseradish peroxidase (HRP) for converting H_2_O_2_ to O_2_ and hydroxyl radicals [38]. To date, ≥40 NMs have been found that exhibit POD-like activity [39]. As shown in Figure 2, 3,3′,5,5′-Tetramethylbenzidine (TMB assay) is a promising protocol for examining materials whether they exhibit POD-like activity or not [39,40]. In the mechanism, NMs can decompose the H_2_O_2_ results oxidation of colorless TMB to colored product (ox-TMB). The overall H_2_O_2_-TMB system process on NMs follows Michaelis–Menten behavior, which strongly confirms the real enzymatic behavior of NMs. Nanozymes have distinct advantages over enzymes, such as high stability, low cost, cyclical use and easy multifunctionalization, making them potential candidates for various environmental and biological applications [41,42]. This review mainly focuses on the biomedical applications of POD mimetic NMs in cancer therapeutics.

### 2.2. Peroxidase Mimetic Nanozymes in Oxygen-dependent Cancer Photodynamic Therapy

PDT is a modern and non-invasive cancer treatment modality [14,43]. As shown Figure 3, in PDT, the first photosensitizer absorbs the incident light of the appropriate wavelength and initiates the activation process [44]. The excited electrons react with the tissue oxygen and generate cytotoxic ROS responsible for cell death. This process shows that, for any efficient PDT effects, three main factors should be considered: (i) the photosensitizer, (ii) appropriate light wavelengths and (iii) dissolved oxygen in cells/tissue. From the type I or type II PDT mechanisms, it is clear that PDT is oxygen-dependent. Unfortunately, oxygen levels in solid tumors are very low, within micromolars (partial pressure of O2 < 5 mmHg corresponding to 7 µM) [45]. This is mainly due to the aggressive proliferation of cancer cells and limited blood supply available to solid tumors. Low oxygen in tumors significantly impacts the efficacy of PDT despite its in vitro potential. Efforts to overcome tumor hypoxia have focused on the production of O_2_ via catalytic reactions [20,46,47], O_2_ carriers and delivery [48,49,50], and O_2_ independent photosensitizers [51,52,53]. However, improved efficacy requires the development of photosensitizers to effectively mediate PDT by addressing tumor hypoxia. To this end, in recent years more efforts have been devoted to developing enzyme mimetic materials, especially POD mimetics, to efficiently overcome tumor hypoxia and mediate the PDT effects converting intracellur/intratumoral H_2_O_2_ to H_2_O and ROS.

**Figure 4:**
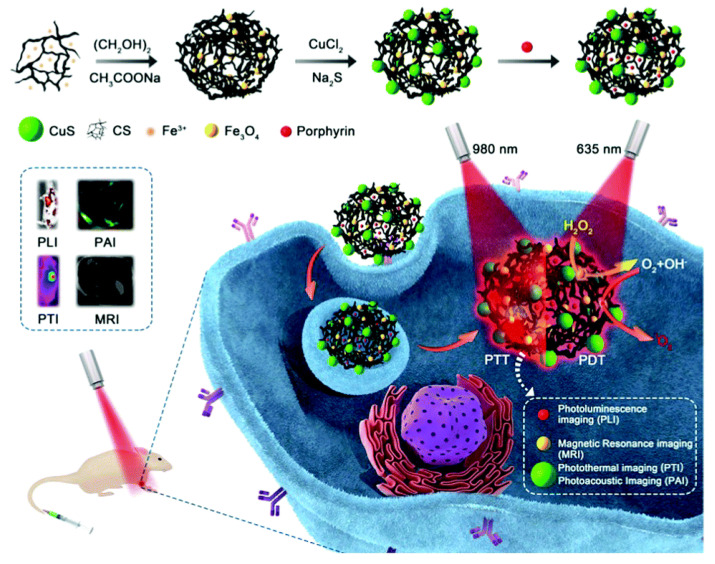
Peroxidaselike Fe_3_O_4_ nanocomposite (FCCP) nanoplatform for photo-induced cancer theranostics. Reproduced with permission from [63].

With the discovery of the enzymatic properties of iron oxide nanoparticles (Fe_3_O_4_ NPs) and iron-based NPs (Au@Fe_3_O_4_ [54], Fe_3_O_4_ coated Ag [55], Pt_48_Pd_52_-Fe_3_O_4_ [56], Fe_3_O_4_@Pt [57], metal organic framework-based MnFe_2_O_4_/C nanozymes, [58], etc.), new attention has focused on bioapplications of peroxidaselike Fe_3_O_4_ nanostructures [38,59]. For instance, cobalt-doped Magnetoferritin (M-HFn) NPs (M-HFn-Co_x_ Fe_3-x_O_4_) with different amounts of cobalt were successfully synthesized. By varying the amounts of cobalt loading into M-HFn cores significantly enhances the peroxidaselike activity and efficacy of visualizing the tumor-specific tissue [60]. Wang et al. reported cobalt-doped Fe_3_O_4_ (Co@Fe_3_O_4_) nanozymes that exhibited stronger peroxidase activity than Fe_3_O_4_ nanozymes alone (100-fold higher affinity) [61]. As a result, Co@Fe_3_O_4_ nanozymes can efficiently catalyze intracellular H_2_O_2_ under low doses, and show promising in vitro and in vivo antitumor efficiency. In another report, An et al. developed a folate conjugated Fe3O4@C NPs which exhibiting peroxidaselike activity. These POD-like activity of NPs significantly promotes ascorbic acid-induced oxidative stress in cancer cells and maximizes the antitumor efficiency [62]. Subsequently, Fe-based nanocomposites were applied to cancer combined phototherapies by overcoming tumor hypoxia via POD-like activities. Zhang et al. fabricated multifunctional chitosan-encapsulated Fe_3_O_4_ nanoparticles modified with CuS and porphyrin (FCCP NPs) for multimodal image guided phototheranostics [63]. As shown in Figure 4, FCCP NPs possess enhanced intrinsic peroxidase mimetic activity to generate ROS from endogenous H_2_O_2_. The in situ generation of ˙OH as a therapeutic agent and provide O_2_ for overcoming resistance to photodynamic therapy results in enhanced in vivo therapeutic efficacy for the treatment of cancer tumors. Notably, Fe_3_O_4_ nanozyme also believed to be CAT activity but authors did not distinguish the difference between the POD-like activity and the CAT-like activity of Fe_3_O_4_ nanozyme. Liu et al. designed and demonstrated a dual enzymelike activities of PtFe@Fe_3_O_4_ nanostructures [64]. Interestingly, PtFe@Fe_3_O_4_ exhibited both the CAT and POD-like activities under acidic conditions which could effectively overcome hypoxia. As a result, successful inhibition of pancreatic cell growth was achieved via photo-enhanced enzyme catalytic therapy. Yang et al. designed a hollow nitrogen-doped carbon nanospheres (HNCSs) and iron phthalocyanine (FePc) for synergistic catalytic and dual phototherapy [65]. FePc/HNCSs simultaneously exhibit POD- and CAT-like activities and facilitates to convert endogenous H_2_O_2_ into ROS and O_2_ for tumor catalytic therapy as well as enhance O_2_-dependent PDT.

**Figure 5:**
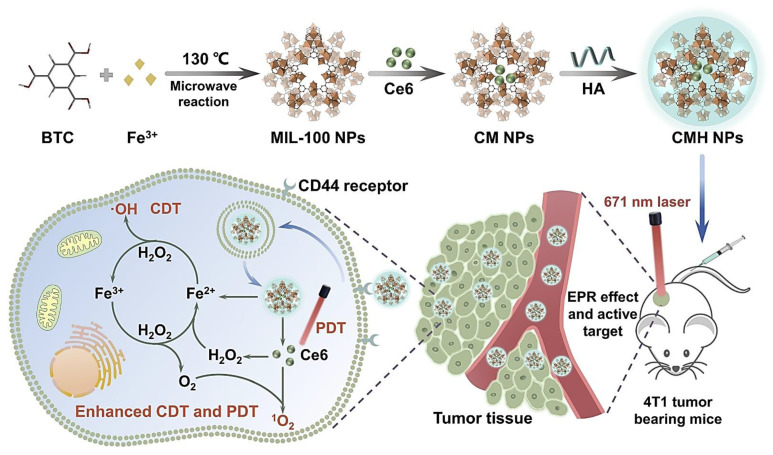
Schematic illustration of CMH NPs for synergetic chemo-photodynamic tumor therapy. Image was reproduced with permission from reference [69].

Aside from Fe-based POD-like nanozymes, other nanomaterials such as platinum (Pt), palladium(Pd), Gold (Au) based nanocomposites, etc. have been examined for use in cancer theranostics. For instance, Cai et al. designed a three-dimensional dendritic mesoporous silica nanosphere (3D-dendritic MSN) material which contains a photosensitizer (Ce6) and Pt NPs for hpoxia overcoming PDT [66]. The incorporated peroxidaselike Pt NPs significantly alleviate the tumor hypoxia by decompostion of intracellular H_2_O_2_ to oxygen species and provide sufficient O_2_ levels to the environment in the tumor for PDT. As a result, this design shows an enhanced PDT effect of killing A549 cells, by overcoming tumor hypoxia. Au NP-doped metal-organic frameworks (GIM) were shown to be potenial candidates to treat hypoxic tumors, where GIM exhibited NIR light-induced glucose oxidase (GOx) activity to generate endogenous H_2_O_2_, which was converted to O_2_ and highly toxic ROS on GIM [67]. Zhang et al. repoted a NIR780 loated serum albumin folate stabilized gold-doped mesoporous carbon (OMCAPs@ rBSA-FA@IR780) nanoprobes as a multifunctional theranostic platform [68]. Besides the therapeutic platform, incorporating Au NPs into mesoporous carbon can facilitate the action of POD mimitic activity and generate ROS for intracellular oxidative damage of cancer cells. As shown in Figure 5, Sheng et al. designed a hyaluronic acid shielded chlorin e6 (Ce6) loaded into a POD mimic metal-organic framework (MOF) MIL-100 nanoparticles (CMH NPs) for synrgetic chemo-PDT [69]. Besides, the generation of cytotoxic singlet oxygen (^1^O_2_) under NIR light irradiation also generates H_2_O_2_. Further, H_2_O_2_ converts to ·OH and O_2_ in a catalytic cascade reactions and alleviates tumor hypoxia. Jana et al. reported a ultrasmall trimetallic (Pd, Cu, and Fe) alloy nanozyme (PCF-a NEs) for ultrasound- and light-enhanced tumor therapy [70]. PCF-a NEs exhibit a cascade POD and GSH peroidase mimitic activities under circumneutral pH. Moreover, photothermal enhanced POD properties on PCF-a NEs facilitate effective tumor cell apoptosis. Most recently, Zeng et al. designed biodegradable POD mimetic boron oxynitride (BON) nanospheres (NSs) for efficient breast cancer therapy [71]. Further, these POD mimetic BON NSs catalytically generate cytotoxic OH radicals for successful inhibition of cancer cells both in in vitro and in vivo.

In addition to the previously mentioned advantages of nanozymes in cancer therapeutics, several further POD mimetic nanozyme platforms such as Au@Co-Fe NPs [72], CuO Nanorods [73], Fe_3_O_4_@MoS_2_-Ag nanozyme [74], Pd nanocrystals [75], and Pt hollow nanodendrites [76], N-doped spongelike carbon spheres (N-SCSs) [77], PEGylated palladium nanozyme (Pd-PEG) [78], tungsten sulfide quantum dots (WS_2_ QDs) [79], nickel disulfide (ND) nanozyme [80], iridium (Ir) nanoplates [81] and MoS_2_ [82] have been successfully utilized in antibacterial applications with significant outcomes. Table 1 summarizes POD-based nanozyme applications in cancer phototherapeutics.

## 3. Current Trends and Future Perspectives

**Figure 6:**
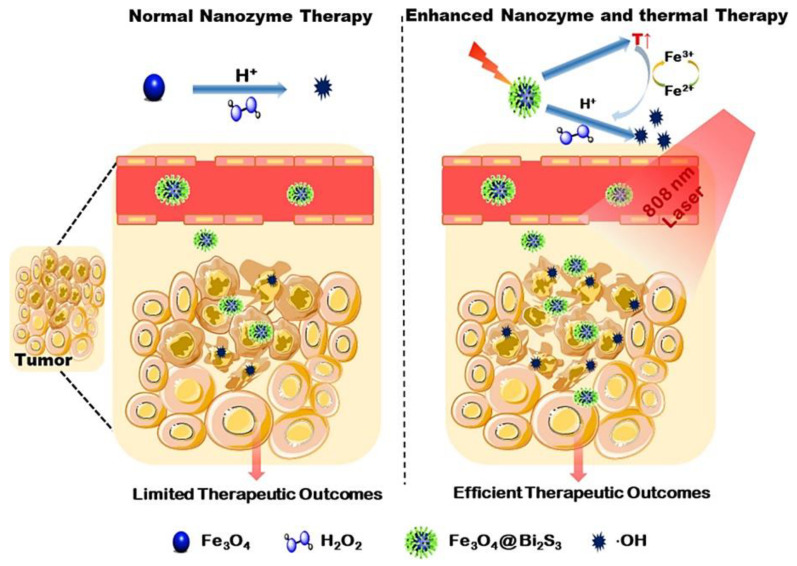
Schematic representation of photothermal-enhanced nanozymatic mediated cancer therapy on viruslike Fe_3_O_4_@Bi_2_S_3_ nanozymes. Image was reproduced with permission from reference [101].

As discussed earlier, several POD mimetic nanozymes have been successfully used to overcome tumor hypoxia. However, the mechanism by which current POD mimetic NMs generate cytotoxic ROS mainly relies on the amount of intracellular H_2_O_2_ and pH. Intracellular H_2_O_2_ concentrations are very low, estimated at around (50 − 100 × 10^−6^ M) [64,99]. As a result, most nanozymes have limited therapeutic efficiency in the tumor microenvironment, and catalytic nanozyme therapy alone is not comparable to combination therapy. To this end, some studies have shown that ROS generation on nanozymes can be improved by photothermal therapy [64,88,100]. Zhang et al. fabricated a viruslike Fe_3_O_4_@Bi_2_S_3_ nanocatalyst (F-BS NCs) by simple ultrasound. Synergistic coupling of POD mimetic Fe_3_O_4_ NPs with a narrow band gap semiconductor Bi2S3 (BS) significantly increased POD mimetic activity [101]. As shown in Figure 6, MNP particles exhibit better enzyme POD-like catalytic activity under mild temperatures compared to at room temperature/no temperature applied. As a result, POD-like activity promotes the conversion frequency of Fe^3+^/Fe^2+^ under mild hyperthermia effect on BS under 808 nm laser irradiation [102,103]. Furthermore, POD enzymatic reaction in the tumor microenvironment improves the yield of ROS and resists the cancer under this mild photothermal effect.

The synergistic effects of dual enzyme mimetic nanostructures in tumor therapy were later investigated. Gao et al. reported a dual inorganic nanozyme-based nanoplatform of Gold (Au) NPs and Fe_3_O_4_ NPs co-loaded mesoporous silica materials for nanocatalytic tumor therapy [104]. Since Au NPs as a GOx mimic, so it will also catalyze β-D-glucose oxidation into gluconic acid and H_2_O_2_, which is subsequently catalyzed by the peroxidase-mimic Fe_3_O_4_ NPs to liberate high-toxic hydroxyl radicals to induce tumor-cell death by the typical Fenton-based catalytic reaction. To further enhance the efficacy of dual nanozyme catalytic therapy, Yi et al. fabricated Wonton-like Bismuth@poly vinyl pyrrolidine@gold platinum (Bi@PVP@AuPt) NPs which exhibited both POD and oxidaselike activity. The stable dual enzymatic behavior of NPs will produce oxygen in hypoxic tumors. Applying the hyperthermia effect to the dual nanozyme significantly promotes ROS generation, resulting in good therapeutic outcomes under combined photothermal and nanocatalytic treatment. Xu et al. designed glucose–oxidase (GOx)-loaded biomimetic Au–Ag hollow nanotriangles (Au–Ag–GOx HTNs) for NIR light-triggered tumor therapy by regulating the tumor environment, where GOx in HTNs triggers the generation of gluconic acid and H_2_O_2_ [105]. Subsequently, H_2_O_2_ will be converted to O_2_ on the POD mimetic HTNs, eventually boosting the formation of •OH radicals under NIR II light for efficient tumor therapy. Dong et al. reported ceria nanozymes decorating uniform Bismuth sulfide nanorods (Bi_2_S_3_ NRs) with dendritic mesoporous silica (Bi_2_S_3_@DMSN) material for tumor catalytic therapy [106]. Synthesized nanozymes exhibited a dual enzyme mimic such as POD and CAT properties under acidic conditions, significantly overcoming tumor hypoxia and elevating oxidative stress under hyperthermia. Recently, Alizadeh et al. reported pH-switchable POD and CAT mimic activities of hierarchical Co(OH)_2_/FeOOH/WO_3_ ternary nanoflowers [107]. The POD activities of the as-synthesized nanoflowers were dominant at acidic pH whereas the CAT activities were dominant at basic pH. Indeed, these catalytic materials produced ROS by decomposition of H_2_O_2_ in both acidic and basic condition, resulting in good anticancer behavior as well as cancer cell detection. Building on these advancements in dual nanozymes and their efficacy in tumor treatment, Ai et al. fabricated a manganese dioxide encapsulated selenium-melanin (Se@Me@MnO_2_) multishell nanozyme for intracellular antioxidation [108]. Se@Me@MnO_2_ nanozyme exhibits multiple enzyme activities such as CAT, SOD and glutathione peroxidase (GPx). This multishell platform can effectively scavenge the ROS species via synergistic and fast electron transfer between Se, Me and MnO_2_. These multienzyme mimetic nanoplatforms are good candidates for future tumor therapy applications.

**Figure 7:**
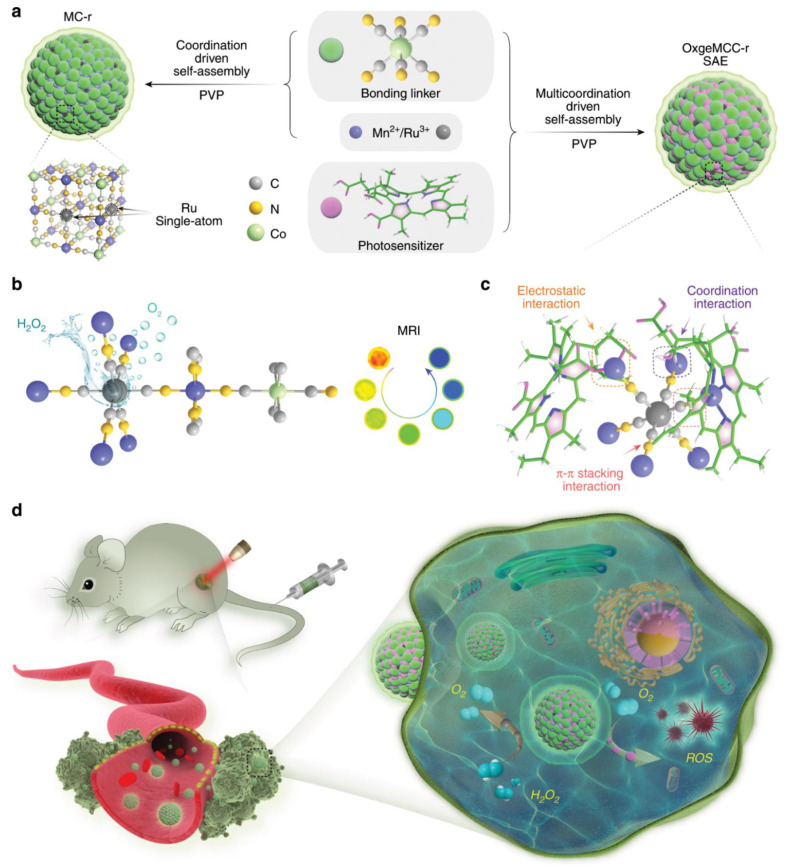
Schematic illustration of Ce_6_ loaded Ruthenium incorporated metal organic framework-based single atom nanozyme (OxgeMCC-r SAE) in PDT. (**a**) Schematic representation of the fabrication of Ru-based single atom nanozyme. (**b**,**c**) are molecular structure and H_2_O_2_ decomposition mechanisms of Ru-based nanozyme. (**d**) Schematic representation of in vivo ROS mediated catalytic therapy on OxgeMCC-r SAE. Image was reproduced with permission from reference [112].

Single-atom-based nanozymes are being developed for various applications. Cost effective and atomically dispersed single atom metal centers can significantly enhance enzyme mimetic properties by maximizing the atomic utilization efficiency and density of active sites. As a result, single atom-based platforms have been developed for various kinds of enzymelike properties such as POD, SOD, CAT, OXD, etc. [109]. By using the enzyme mimetic properties of single atoms, Xu et al. fabricated a zinc-based single atom supported by a metal organic framework and observed excellent POD-like properties [110]. These POD-like activities further help to efficiently deactivate in vivo bacterial infections. Huo et al. fabricated PEGylated single-atom Fe-containing nanocatalysts (PSAF NCs) atoms and observed that the present Fe-based single atom could effectively trigger intracellular H_2_O_2_ and selectively generate hydroxyl radicals (•OH) in acidic tumor environments [111]. More recently, Wang et al. fabricated single-atom ruthenium as the active catalytic site anchored in a metal-organic framework Mn_3_[Co(CN)_6]2_ with encapsulated chlorin e6 (Ce_6_), which serves as a catalaselike nanozyme for oxygen generation [112]. Figure 7 presents a schematic representation of the detailed fabrication and in vivo applications. Single-atom Ru loading content can degrade intracellular H_2_O_2_ to O_2_ to relieve hypoxia in solid tumors, leading to enhanced ROS generation, and finally causing apoptotic cell death both in vitro and in vivo.

Despite nanozymes offering significant advantages such as high stability, low cost, long-term stability and large scale production, several considerations/improvements are needed for future practical applications. Some key considerations for future nanozymes in cancer applications are as follows.

(i) Activity: Most nanozymes exhibit lower activity than natural enzymes, possibly due to the additional surface conjugation on NMs, thus, the development of new surface conjugation strategies to improve nanozyme activity is highly desired.
(ii) Sensitivity: Most nanozymes show good performance in in vitro studies but their application in biomedical applications is still questionable. For example, POD mimetic nanozymes in cancer therapy were designed to alleviate tumor hypoxia, but the presence of in vivo endogenous H2O2 is at concentrations of a few micromolars, thus, nanozymes must be ultra-sensitive to detect and decompose the H_2_O_2_ to O_2_.
(iii) Toxicity/biosafety: Unlike natural enzymes, the cytotoxicity effects and biocompatibility of nanozymes are still unconfirmed. Therefore, more research is needed prior to the development of practical applications.
(iv) Selectivity: Most nanozymes can catalyze a broad range of substrates for multiple enzyme activity. For example, some enzymes exhibit both POD and CAT or POD and OXD mimetic activities. Although several studies have examined various types of surface conjugation techniques to attain selectivity, a complete investigation of catalytic mechanisms is still needed.
(v) Theoretical studies: Additional theoretical studies of nanozymes are needed to combine both experimental and computational results to better understand their complete mechanisms of nanozymes.
(vi) Limited to cancer therapy: Currently, most nanozymes are limited to cancer therapy. Nanozyme-mediated disease diagnostics and therapeutics should be applied to other diseases as well as the environmental and agricultural domains.
(vii) Single atoms: Single atom nanozymes represent potential candidates for cancer therapeutic applications. However, more studies are needed on long term cytotoxicity, biosafety, stability and mechanisms.
(viii) Most nanozymes are limited to only POD, CAT, OXD and SOD. Future work should explore nanozymes in terms of other enzyme mimetic activities for use in a wide variety of applications.

Besides a good design of POD nanozymes, one should also consider the current objective evaluation indicators in tumor treatment for significant future therapeutic improvements. By addressing all the concerns, the design/fabrication of novel and efficient nanozymes will show considerable potential for a broad range of research for future environmental, agricultural and biomedical applications.

## 4. Conclusions

In summary, this review presents past and current advancements in the development of nanozymes, especially POD mimetic nanomaterials for oxygen-dependent phototherapy, with a detailed explanation of the mechanisms and roles of POD mimetic nanozymes in cancer therapeutics. The main obstacle of effective tumor phototherapy is tumor hypoxia. Most phototherapy modalities are oxygen-dependent, and tumor microenvironments contain insufficient oxygen for effective therapeutic application. To overcome this limitation, we review the advantages of POD mimetic nanomaterials which can catalyze endogenous H_2_O_2_ to H_2_O and ROS, thus overcoming tumor hypoxia. Further therapeutic improvement can be achieved through combining therapeutic platforms such as mild PTT-induced enhancement of nanozyme activities and dual, multienzyme strategies. Future perspectives and challenges facing the continued development of nanozyme applications are discussed to elucidate directions for future nanozyme-based therapeutics and to transform clinical settings. We believe the present review helps to provide a more systematic understanding of the advantages, mechanisms and challenges of nanozymes and will facilitate the development of novel POD mimetic nanozymes for efficient cancer phototherapy applications.

## Figures and Tables

**Table 1:** Recent reports on POD-based nanozyme-mediated cancer phototherapeutics.

S. No.	Nanozyme	Enzyme Mimetic Properties	Application	Ref.
1	Au2Pt-PEG-Ce6	POD, CAT	Chemodynamic therapy/phototherapy	[83]
2	SFO nanozyme	POD, CAT	Synergistic phototherapy	[84]
3	Cu_2_MoS_4_ (CMS) loaded with GOx	CAT and GPOD activities	CDT/starvation/photo/immunotherapy	[85]
4	ZIF-8 NPs coated with Ce6 and Cyt c	POD, CAT	PDT and protein therapy	[86]
5	Au-Ag@HA NP	POD	Radiation/nanozyme/Ag+ combined therapy	[87]
6	Au@HCNs	POD and OXD	Enzyme catalytic-PTT	[88]
7	ABTS@PAH-CNts	POD	PTT	[89]
8	PB-Ft NPs	POD	Chemo-PTT via ROS production	[90]
9	PCN-224-Pt	POD, CAT	PDT	[91]
10	AgPd@BSA/DOX	POD	ROS/hyperthermia/chemotherapy	[92]
11	Fe^ΙΙΙ^-doped C_3_N_4_ nanosheets	POD	MRI guided PDT	[93]
12	MIONzyme-GOx	POD and GOx	ROS induced damage	[94]
13	PDAC NPs	POD	PTT/CDT/CHT combination therapy	[95]
14	GQD-SPNs	POD	PTT enhanced cancer catalytic therapy	[96]
15	FeN200@GOx@M	POD	combinational therapeutic approach (UTMD and enzyme)	[97]
16	Co_9_S_8_ NDs	POD	PTT/PDT	[98]

Abbreviations: chlorin e6 (Ce6); polyethylene glycol (PEG); SnFe2O4 (SFO); Au-Ag@HA NP; gold nanoparticle core with a porous hollow carbon shell nanospheres (Au@HCNs); PEGylated CMS@GO_x_; zeolitic imidazolate framework-8 (ZIF-8); dual lock-and-key type activatable nanotherapeutic platform (ABTS@PAH-CNts), 2,2′-azino-bis(3-ethylbenzothiazoline-6-sulfonic acid) (ABTS); ceria nanocubes (CN); Prussian blue-modified ferritin nanoparticles (PB-Ft NPs); bovine serum capped bimetallic silver palladium nanoparticles loaded with doxorubicin (AgPd@BSA/DOX); iron oxide-based nanozymes loaded with glucose oxidase (MIONzyme-GOx); polydopamine (PDA) and ammonium bicarbonate (NH4HCO3) coated and doxorubicin (Dox) loaded hollow cerium oxide (CeO_2_) NPs (PDAC NPs); graphene quantum dots/semiconducting polymer nanocomposites (GQD-SPNs); ultrasound-targeted microbubble destruction (UTMD); chemotherapy (CHT); Fe-Metal organic framework-based nanozyme (FeN); chemodynamic therapy (CDT); cobalt sulfide nanodots (Co_9_S_8_ NDs).

## Data Availability

Not applicable.
